# Towards Curative Therapy in Burkitt Lymphoma: The Role of Early African Studies in Demonstrating the Value of Combination Therapy and CNS Prophylaxis

**DOI:** 10.1155/2012/130680

**Published:** 2012-01-11

**Authors:** Ian Magrath

**Affiliations:** ^1^International Network for Cancer Treatment and Research, Rue Engeland 642, 1180 Brussels, Belgium; ^2^Uniformed Services University of the Health Sciences, Bethesda, MD 20814, USA; ^3^National Cancer Institute, Bethesda, MD 20892, USA

## Abstract

This paper describes the treatment of Burkitt lymphoma (BL) from the time of its discovery in Africa up to the present. Pioneer investigators explored the value of chemotherapy since surgery and radiation were not effective modalities. Complete response was observed with many drugs used as single agents, but Ziegler and colleagues showed that patients resistant to one drug could achieve cure and potentially long-term survival with other drugs. Subsequently, a combination of cyclophosphamide (CTX), vincristine (VCR), and methotrexate (MTX) was shown to be active, but a survival advantage compared to CTX alone could not be demonstrated because effective CNS prophylactic therapy, in the form of intrathecal therapy, was not given. A recent re-evaluation of this regimen in Africa with multiple doses of intrathecal therapy compares favourably with recent studies of single agent CTX, and other drugs have been shown to be non-cross resistant. Optimal results for patients with extensive disease probably require 5 or 6 effective drugs along with intrathecal therapy, using MTX and Ara-C. In Africa, doses must be lower, because of limitations in supportive care, but in technically advanced countries cure rates in excess of 90% can be obtained. Rituximab may improve the results in some patient groups and allow less intensive therapy without a reduction in survival in others.

## 1. Introduction

Burkitt lymphoma (BL), in spite of its low incidence throughout most of the world, has had a major impact upon the understanding and treatment of lymphomas and doubtless upon many other cancers. The fact that it was discovered in equatorial Africa and much of the groundwork for the evolution of its treatment was conducted in equatorial Africa in the 1960s and 70s, but little since, is an indication of the great loss to research that results from the enormous differences in resources that exist among the world's countries. The rich variety of lifestyles, diets, and environmental exposures in the world have probably never been greater, yet the vast bulk of research is conducted in the high income countries and in particular the OECD countries. Much of this research is not relevant, at least, in the immediate future, to the low- and many middle-income countries, which have not yet built strong enough infrastructures to carry out their own scientific investigations. Moreover, in the realm of treatment, regimens have constantly increased in complexity and cost with often questionable advantage, certainly when seen from the perspective of population medicine. Classification and staging systems become more and more complex such that diagnosis and staging comprise significant elements of the cost of managing a patient, without necessarily resulting in improved survival. In the case of Burkitt lymphoma, Burkitt himself showed many years ago that a small fraction of patients could be cured with a single dose of chemotherapy and that the extent of disease was perhaps the most important determinant of outcome, yet while patients with limited disease do receive limited treatment, much of the more recent research into the treatment of patients with extensive disease has been focused on determining which elements of very intensive, albeit successful therapies devised in recent years, can be removed with impunity. Such studies have revealed a number of superfluous elements, indicating that the first highly successful therapies were also excessively and, doubtless, therefore, more toxic and more expensive. This is a very different approach from that taken by pioneer chemotherapists in Africa, where it was felt that the host response provided an important part of therapy, and too much chemotherapy could, by abrogating immunological defences against the tumor, lead to a negative result. Unfortunately, this approach often led to patients being undertreated, and since every patient is different, precisely how intensive the management of patients needs to be is not easy to determine. Nonetheless, today, most patients with BL can be cured with intensive conventional chemotherapy by accepting the fact that some will be over-treated, but directing research towards trying to identify such patients and minimizing therapy to the extent possible. This approach has led to approximately 90% of children, and a somewhat lower percentage of adults, being cured of their disease.

With the advent of highly active antiretroviral therapy, it has proved possible to use intensive therapy in AIDS patients, with a similar outcome to that achieved in non-AIDS patients. In Africa, there should be little difficulty in achieving reasonable survival rates in AIDS-related BL as long as patients also receive HAART, although the disease burden is likely to remain an important determinant of outcome.

## 2. Discovery of the Tumor and Early Therapeutic Studies

Dennis Burkitt was a surgeon who had been practicing at the Mulago Hospital in Kampala, Uganda for almost ten years before he saw his first case of the lymphoma which subsequently bore his name. Burkitt had reported a clinical syndrome of jaw tumors with or without tumor at other sites (particularly the abdomen) in 1958 [1]. Shortly afterwards, O'Conor and Davis published a review of childhood cancer in Uganda in which they reported that approximately 50% of all malignant diseases in children had similar pathology with clinical features consistent with those reported by Burkitt, although not all had jaw tumors. They identified the disease as a poorly differentiated lymphocytic lymphoma [2]. Surgery had little to offer these children, and radiation therapy was not then available in equatorial Africa, but, in 1960, David Burchenal, who had been using methotrexate (MTX) in patients with leukemia at the Sloane Kettering Institute in New York, visited Mulago Hospital, bringing some of the drug with him. Burkitt was persuaded to try the drug in two children. Both had remarkably rapid responses after a single dose of MTX, and although the first child subsequently relapsed, the second had a prolonged remission. In the pioneering days of chemotherapy, when many skeptics viewed cytotoxic therapy as merely a means of delaying the inevitable, this was a critically important result and triggered a great deal of interest in further treatment studies. In the course of the next several years, Burkitt in Uganda, Clifford and Oettgen in Kenya, and Ngu in Nigeria, assisted by collaborators in the USA and UK and drug donations, examined responses to many of the drugs then available (Table 1) [3–7]. This was a less than systematic process, and the drugs were used in various doses, schedules, and even routes of administration, but given the absence of any prior data on treatment response, the demonstration of clear diminution in tumor size, complete clinical remission, and over time, prolonged survival, even, on occasion, with single doses of drug, clearly demonstrated the high degree of chemosensitivity of the African lymphoma [8–11], particularly, but not exclusively, to cyclophosphamide (CTX), methotrexate (MTX), and vincristine (VCR) [3–8]. Anthracyclines (initially daunomycin) were not developed until the second half of the 1960s so were not assessed in these early studies. Indeed, single agent data is not available for the anthracyclines although they were informally given to some patients who relapsed (daunomycin or hydroxydaunomycin) with minimal responses being seen, such that these drugs were not further pursued at that time. Interestingly, in contrast to the approach to acute lymphoblastic leukemia in, for example, the USA, and related to both the extraordinarily rapid response to therapy and difficulty for African patients to stay at or near the hospital for prolonged periods, most patients were treated with only one or two doses of the drug being tested—in retrospect, sufficient to cure only a relatively small fraction of patients, mostly with limited disease.

In Burkitt's series of 90 jaw tumors treated at the Mulago Hospital Uganda with CTX, MTX, or VCR [9], 82% had complete (CR) or partial responses (PR), those with small tumors being much more likely (10 of 10) that those with large tumors (10 of 40) to achieve a CR, suggesting a relationship between response and tumor size, which was subsequently confirmed [10, 11]. Indeed, it seems likely that the extent of tumor is the single most important determinant of treatment outcome, since even patients with central nervous system disease (CNS) can be cured, particularly when the total tumor burden is small—a situation relatively common in Africa, where many patients with CNS disease have either a jaw or orbital tumor, or a small extradural mass, all of which create major symptomatology and therefore early diagnosis—but rare elsewhere, where CNS disease is usually associated with extensive disease, particularly bone marrow involvement. Burkitt also pointed out that complete remissions occur within a few weeks or not at all and that relapse is very uncommon after a year of remission. Such “very late” relapses may, in fact, be second, clonally discrete tumors occurring in patients at very high risk for BL [13].

Longer followup in Uganda suggested that approximately 20% of patients could be expected to achieve long duration remissions after (by today's standards) minimal therapy [14–16], and Burkitt noted that recurrence could occur at previously uninvolved sites, a finding later confirmed and expanded by Ziegler, who reported that early relapses (within 10 weeks of first treatment) were more likely to recur at the original site of disease and/or in the central nervous system (CNS), while those with late relapse (after 10 weeks, and with a median remission duration of 26 weeks) were more likely to relapse at previously uninvolved sites of disease and only rarely in the CNS. The latter patients were also much more sensitive to further chemotherapy than the former [17, 18]. Burkitt noted that, in 12 patients known to have died after a complete or almost complete remission, 6 were due to CNS lesions—at that time, no preventative intrathecal chemotherapy was given to any of the patients.

Ngu at the University College Hospital in Ibadan, Nigeria, and Clifford, at the Kenyatta Hospital, in Nairobi, also reported dramatic responses and long-term survivors after treatment with CTX as well as, in Clifford's hands, melphalan or orthomelphalan [4, 7, 8, 15], although patients with extensive disease rarely achieved long-term survival. Ngu brought attention to the fact that serum uric acid levels were often raised in patients with extensive tumors and sometimes became even more elevated following therapy, in which case oliguric renal failure and death from electrolyte disturbances were the result. Thus, Ngu appears to be the first to describe what would now be called the acute tumor lysis syndrome (serum uric acid on the day of death was 54 mg per 100 mL) and reported that this and other complications such as perforation of the bowel may ensue from rapid dissolution of tumor following therapy. Knowledge of the severe consequences of untreated massive tumor lysis syndrome led to approaches to its management—initially massive hydration with allopurinol to ensure that the tumor-derived uric acid load was excreted. Alkalinization can be used during this phase, since uric acid is more soluble in an alkaline urine. But care should be taken not to “overshoot” since once the uric acid level is sufficiently low, the major problem becomes excretion of phosphate, which is less soluble in an alkaline urine and if insufficient fluids are given will precipitate in the renal tubules, causing obstructive, oliguric renal failure which is rapidly fatal in the absence of hemodialysis or filtration, which was not then, and is rarely now, available in most hospitals in low income countries. Thus, the key to the management of tumor lysis syndrome in patients with high tumor burdens is hyperhydration, allopurinol, to decrease uric acid formation and allow more oxypurine to e excreted as xanthine and hypoxanthine with careful observation of the patient to ensure that a good urine flow is sustained.

At this time, the relationship between BL and acute lymphoblastic leukemia, a rare disease in equatorial Africa, was frequently debated [19]. Clift et al. described a group of 4 children with cytologically typical BL who had a leukemic blood picture and/or diffuse bone marrow involvement at either relapse or presentation [20] as rarities—overt leukemia was rarely seen in African patients and although bone marrow examinations were infrequently performed in life, bone marrow involvement was present in less than 10% of children who died from BL [21, 22]. Figures on incidence of acute lymphoblastic leukemia (ALL) remain imprecise, but it does seem to be becoming more frequent, as one would expect, particularly in urban regions. Two of the children reported by Clift were initially treated with prednisone (40 mgs per day) and mercaptopurine, then standard therapy for acute lymphoblastic leukemia (ALL). Neither responded but one of the children was subsequently treated with nitrogen mustard and there was marked, although temporary, tumor regression [19]. Many years later, a comparison in children of ALL-like therapy versus repeated courses of a drug combination including CTX, MTX, VCR, and prednisone, carried out in the USA, showed a clear advantage of the latter regime in patients with BL [23], although the role of prednisone is unknown [20]. Similarly, anthracyclines have not been tested as single agents in first-line therapy of BL, but are included in most treatment regimens because of their successful introduction into the therapy of lymphomas in adults. It remains possible that they add little but toxicity to treatment regimens for BL.

### 2.1. Non-Cross-Resistant Drugs

In the late 1960s and early 1970s, a collaboration between the Uganda Cancer Institute (UCI) of Makerere University in Kampala and the National Cancer Institute (NCI) of the USA resulted in several other important observations. Of particular importance were the demonstrations that CTX, VCR, and MTX were in large part non-cross-resistant. Comparison of a single dose of CTX with 6 doses (patients who achieved CR after one dose being randomized to no more CTX or 5 more doses) [10] showed that, among stage III patients treated with a single dose of CTX, relapses occurred in the same site as the original tumor, sometimes with concomitant CNS disease and nearly always within 2 to 8 weeks of randomization. Such patients responded to further CTX, 7 of 7 achieving a second CR, indicating that a single dose of CTX represented, for the vast majority of patients, inadequate therapy. Stage III patients randomized to receive multiple doses of CTX tended to relapse after 3–5 doses of CTX and all failed to respond to additional CTX. Ten of the total of 24 patients who relapsed after a CR following single-agent CTX, either during or shortly after the completion of therapy were treated with a combination of VCR and MTX followed by Ara-C, a regimen known as BIKE. Nine achieved a CR, 8 remaining free of disease for 30–102 weeks (i.e., most were probably cured). These data suggested that drug combinations would be likely to result in improved treatment outcome. This was an important finding.

### 2.2. Early Drug Combinations

The efficacy of combination chemotherapy was tested in a second clinical trial conducted at the LTC in which patients with stage III or IV disease, who were randomized at the time of presentation to receive either 6 doses of cyclophosphamide (40 mgs/kg every 2-3 weeks—24 patients) or a sequential combination regimen (TRIKE) consisting of CTX followed by VCR and MTX followed by Ara-C (18 patients). These therapy components were administered at approximately 2 weekly intervals until 2 cycles of TRIKE had been given [24]. Although the overall relapse rate between the two arms of the study were quite similar (with also no difference between stage III and IV patients), there was a trend in favor of TRIKE. Patients who received CTX alone invariably recurred at the same anatomical location (often with CNS disease), and 7 of 8 who relapsed on treatment failed to respond to continuation of CTX. In contrast, relapse in the TRIKE arm tended to occur earlier, and, of 9 patients who relapsed on therapy, 6 responded to continuation of TRIKE (involving different agents), consistent with the notion that the drugs were non-cross-resistant. Since at least some of the patients treated in the CTX alone arm could be salvaged with BIKE and because of the small numbers of patients available for comparison, there appeared at first to be no clear advantage in terms of overall survival, although later followup did indicate a survival advantage to patients initially treated with TRIKE [25], again consistent with the value of combination chemotherapy.

The early results achieved in the TRIKE trial suggested that the use of a simultaneous drug combination from the beginning would be advantageous. Accordingly, a randomized study was performed in which patients were initially treated with CTX alone (2 doses 2 weeks apart) or with two cycles of COM (a combination of CTX, VCR, and MTX repeated after two weeks) [26]. Since an effective means of preventing CNS disease had still not been identified, no IT therapy was given unless CNS disease was present (at presentation or relapse). However, patients who achieved remission were randomized to receive no further therapy or craniospinal irradiation in Nairobi, where a radiation therapy unit had recently been established. A dose of 20–24 Gy, in 30 fractions over two weeks, was given, but this dose failed to prevent CNS recurrence [27] and did not influence the outcome of therapy. The proportion of relapses treated with CTX alone or with COM was similar over the follow-up period, but 7 of the 8 recurrences among 19 patients treated with CTX alone involved both systemic and CNS disease, while 8 of the 10 recurrences in the 21 patients who received COM were confined to the CNS. Later followup indicated a survival advantage for patients treated with COM from the outset [25], but the study also emphasized, once again, the need for effective prevention of CNS spread. Although inadequate by modern standards, these data all pointed towards combinations of drugs being more effective, but, in the absence of effective CNS treatment, their advantage could not be realized.

In a similar time period, Nkrumah and Perkins, conducted a series of studies in Accra, Ghana, initially supporting the conclusion that the BIKE regimen was non-cross-resistant and also that simultaneous combination therapy (in Ghana, a combination of CTX, Ara-C, and vincristine was used, along with intrathecal methotrexate, during each course of therapy) was likely to be more effective [28, 29]. Very few patients developed CNS relapse compared to a previous group of patients treated with CTX alone, and the frequency of systemic relapse was also much lower in patients randomized to combination therapy. Unfortunately, the high toxic death rate in the latter arm prevented the demonstration of a survival advantage. This study did suggest, however, that the reason that intrathecal therapy had not been effective previously is that it was not sufficiently prolonged—one cycle is not enough.

### 2.3. Prevention and Treatment of CNS Disease

Further confirmation of the need for multiple cycles of CNS therapy had been suggested by early studies at the LTC in which IT chemotherapy was given only to patients with overt CNS disease. Various regimens, ranging from single weekly doses to (remarkably) 10 consecutive days of therapy with either methotrexate or Ara C, had been used but little success was achieved. In contrast, a fraction of patients with CNS disease at the time of relapse—for example, almost a third of those with isolated CNS relapse—achieved long-term survival when given multiple doses of IT therapy in conjunction with additional systemic therapy. Moreover, some patients with CNS recurrence after intrathecal methotrexate had durable remissions when treated with multiple-dose IT Ara-C [30], indicating the value of both drugs and suggesting the need for more than a single course of therapy for effective CNS prophylaxis. Such a conclusion is taken as self-evident today, although it did not seem obvious at that time. Remarkably, however, (approximately 50%) of patients with CNS disease occurring at any time in their disease achieved prolonged survival following multiple doses of intrathecal therapy, again, entirely consistent with the likelihood that many patients were simply being inadequately treated with intrathecal therapy.

Interestingly, unlike BL in the USA and Europe, a significant fraction of patients presenting with CNS disease in Africa have limited disease—for example, a jaw tumor or isolated CNS relapse and many achieved prolonged survival, although patients who had simultaneous CNS and systemic relapse had a worse outcome [31]. This strongly suggested that total tumor burden was more important than the mere presence or absence of CNS disease in predicting outcome. In contrast, patients in North America or Europe, where CNS disease tends to occur in patients with systemically advanced disease, particularly when there is bone marrow involvement, have a poor prognosis—doubtless primarily due to the overall disease burden, but interpreted for some years as indicating that CNS disease *per se* was a poor prognostic indicator—its presence is still sufficient to classify a patient as stage IV [32].

### 2.4. The Role of Other Therapeutic Modalities

Chemotherapy constitutes the primary therapy for BL. Surgery may have a limited role in countries where sufficiently intensive therapy is not available—for example, when patients have access only to CTX, but very few patients, apart from those in rural Africa fall into this category. A retrospective study conducted in Uganda showed a survival advantage to patients in whom the bulk (estimated to be at least 90%) of all abdominal tumor was resected prior to the commencement of chemotherapy [33]. Surgery may be disadvantageous in patients who are to receive high intensity therapy but, in patients presenting with insussusception due to localized small-volume bowel disease, total resection and limited chemotherapy, without intrathecal therapy, results in an almost 100% survival rate.

Radiation therapy was also studied in Africa after radiation became available in Nairobi. Single daily dose radiation therapy was shown to be quite ineffective for the control of local disease (with remission occurring in only 1 of 9 irradiated tumors), although hyperfractionation appeared to be of some benefit [34]. No new evidence contradicting these data has been produced, either in Africa or elsewhere, and radiation is not a component of standard therapy for BL. These findings are very important to equatorial Africa, for the rare need for surgery, and poor results of radiation therapy indicate that this most common of pediatric cancers requires little more than expert knowledge, a few drugs, and a minimum of equipment for patients to be successfully treated. This should not be interpreted to mean that patients can be treated at any district hospital. It is essential that the treating physician is familiar with the characteristics of the disease, the side effects, and the complications that can arise from therapy and that there are adequate facilities for hydration—and if necessary dialysis. 

## 3. The Evolution of Therapy in the USA, Europe and Adults

The COM regimen was adopted at the US National Cancer Institute in the 1970s [35] and formed the backbone on which modern intensive chemotherapy regimens used firstly in children with BL and subsequently in adults [36–41] have been developed. The regimens have become increasingly intensive and have led to survival rates of approximately 90%. In fact, the primary goal of therapeutic studies today is to define risk groups as accurately as possible, such that patients receive the minimum of therapy consistent with the best possible outcome. Initially, these more intensive protocols were more intensive than they needed to be, and it has taken some years and a number of studies to demonstrate this. It is even possible that anthracycline, added when this drug was felt to be of major importance in the treatment of adult non-Hodgkin's lymphoma, adds nothing but toxicity to childhood Burkitt lymphoma. Late cardiac toxicity occurs at much lower cumulative doses than acute toxicity and is a particular problem in children whose cardiac development may be impaired and should, if at all possible, be avoided. A randomized study is currently being conducted to explore this. In addition, the advent of rituximab has led to the possibility of obtaining the same excellent results with less toxicity. Once again, this drug is being tested in children with Burkitt lymphoma, although the excellent results achieved with modern intensive chemotherapy mean that, initially, subgroups of patients with a somewhat worse prognosis must be identified for clinical trials of the efficacy of ritixumab, or use made of phase II windows [42]. One such study did show efficacy of rituximab, and, in adults, where the outcome of therapy has been generally somewhat worse than in children, it has been simpler to add ritixumab to therapy and demonstrate an advantage [43, 44]. 

The intensive treatment approaches used in the western world are not suitable for use in equatorial Africa, where facilities for supportive care remain extremely limited—the paucity of appropriate treatment facilities coupled to poverty have a marked impact on access to care. Recently, therefore, several groups, including the International Society of Pediatric Oncology, the Groupe Francophone pour Oncologie Pediatrique, and the International Network for Cancer Treatment and Research, have worked with colleagues in Africa and other low- and middle-income countries to develop suitable protocols for African countries (including North Africa, where facilities are better) [45–48].

### 3.1. Therapy of African Burkitt Lymphoma Today

An initial study in Malawi with therapy based on the French LMB studies but much less intensive, in which patients with St Jude stage IV were excluded, showed that event-free survival was 57% for all patients (10 stage I, 5 stage II, and 29 stage III) with a stable survival curve at 500 days [45]. Cyclophosphamide monotherapy was also retrospectively utilized in Malawi in the late 1990s at a dose of 40 mgs per Kg every two weeks. Patients received between 1 and 12 doses [46]. Only 72 of 90 patients were evaluable, but the possibility of cure of patients with limited facial disease using therapy was confirmed (63% survival in patients with disease in the head only), and some patients with more extensive disease also survived (33% survival in 21 children with tumor involving the abdomen or other sites).

In 2000, a SIOP study was initiated in Malawi consisting of a combination of cyclophosphamide (O), vincristine (V), prednisone (P), and methotrexate (M) given as COP1, COP2, COMP1, and COMP2 [47]. A total of 30 boys and 12 girls were entered in study, but 14 patients died during or shortly after the initiation of therapy, and projected event-free survival at one year is not promising, being 50% in stage I and II, 33% in stage III, and 25% in stage IV (33% overall). Although morbidity and mortality was high, only 300 mgs of cyclophosphamide was used, being increased to 500 mgs in the COMP cycles. The authors felt that the likeliest explanation for the poor results was the low cumulative dose of cyclophosphamide. A small protocol (40 patients) of higher-dose cyclophosphamide (40 mgs per M2), IT methotrexate, and hydrocortisone conducted in Malawi in 2006 gave a result of approximately 48% survival for an average cost of less than $50 [49]. The ability to “rescue” patients with high-dose cyclophosphamide and methotrexate [50], suggests that many of these patients, as in the 1960s and 1970s, were undertreated.

A much larger study being conducted by the INCTR in several equatorial African countries is in progress, and initial results look promising. This protocol uses higher doses of cyclophosphamide (1200 mgs/m^2^) with vincristine and methotrexate as the first-line therapy, and has a salvage regimen (second line), which has induced complete responses in approximately a third of patients resistant to the first-line therapy. This approach, in which future higher risk patients will receive both the first- and second-line therapy (rituximab remains a possibility but may have to be excluded because of cost), seems likely to produce very acceptable results for equatorial Africa as it already has in India, Egypt, and the USA, albeit with higher doses in the USA [40, 41, 51, 52].

In countries where resources are less limited, results are better since more intensive therapy can be safely given to patients with extensive disease; for example, somewhat modified versions of the highly effective French and German protocols. Depending upon the level of development, results approach those being achieved in high-income countries. Sadly, therefore, one is forced to conclude that a large number of children in equatorial Africa still receive inadequate therapy, and outside formal clinical trials, the advances made 30–40 years ago (for the most part) [53, 54] have led to only limited gains, in the ensuing period in terms of the cure of patients with African Burkitt lymphoma, largely because of the major resource limitations and poor access to care.

## 4. Conclusions

African BL, discovered and initially investigated in equatorial Africa, has provided an important model for the epidemiology, pathogenesis, and treatment of many other lymphomas. In particular, the value of combination therapy and intrathecal prophylaxis were strongly suggested in the early clinical trials in African Burkitt lymphoma although numbers were small. Collaboration between resource-rich countries and African countries should both improve the care of patients with BL in Africa, where treatment needs to be intensified, and lessen the side effects of therapy outside Africa, where treatment is probably still more intensive than it need be. The use of newer agents such as rituximab may prove to be prohibitively expensive for the time being but is likely eventually to be introduced into the treatment of African patients for reasons of lower toxicity and, eventually, overall lower cost (the treatment of toxic complications can be costly).

## Figures and Tables

**Table 1:** 

Drug	No. of patients	CR	CR + PR	%OR
Cyclophosphamide	163	43	132	81
Orthomerphalan	14	UK	14	100
Chlorambucil	12	3	10	83
Nitrogen mustard	61	10	44	72
Melphalan	26	8	16	61
Procarbazine	6	0	0	0
BCNU	5	0	4	80
Vincristine	21	10	17	81
Vinblastine	2	0	0	0
Methotrexate	45	11	26	58
6-Mercaptopurine	3	0	0	0
Cytosine arabinoside	3	2	2	2
Epipodophyllotoxin	2	2	2	2
Actinomycin D	4	1	4	4

CR: complete response; PR: partial respo2nse; OR: overall response.

Various doses and schedules were used, even for the same drug.

Adapted slightly from [12], UK = unknown.

## References

[1] Burkitt D (1958). A sarcoma involving the jaws in African children. *The British Journal of Surgery*.

[2] O’Conor GT, Davies JNP (1960). Malignant tumors in African children. With special reference to malignant lymphoma. *The Journal of Pediatrics*.

[3] Oettgen HF, Burkitt D, Burchenal JH (1963). Malignant lymphoma involving the jaw in African children: treatment with Methotrexate. *Cancer*.

[4] Oettgen HF, Clifford P, Burkitt DP (1963). Malignant lymphoma involving he jaw in African children. Treatment with alkylating agents and Actinomycin D. *Cancer Chemotherapy Reports*.

[5] Burkitt D, Hutt MS, Wright DH (1965). The African lymphoma: preliminary observations on the response to therapy. *Cancer*.

[6] Burkitt D (1966). African lymphoma. Observations on response to vincristine sulphate therapy. *Cancer*.

[7] Ngu VA (1972). Chemotherapy of Burkitt’s tumor at the University of Ibadan, Nigeria. *Journal of the American Medical Association*.

[8] Ngu VA (1965). The African lymphoma. (Burkitt tumor). Survivals exceeding two years. *British journal of cancer*.

[9] Burkitt D, Burchenal JH, Burkitt DP (1967). Chemotherapy of jaw tumours. *Treatment of Burkitt's Tumour*.

[10] Ziegler JL, Morrow RH, Fass L, Kyalwazi SK, Carbone PP (1970). Treatment of Burkitt’s tumor with cyclophosphamide. *Cancer*.

[11] Magrath I, Lee YJ, Anderson T (1980). Prognostic factors in Burkitt’s lymphoma. Importance of total tumor burden. *Cancer*.

[12] Magrath IT (1991). African Burkitt's lymphoma: history, biology, clinical features, and treatment. *American Journal of Pediatric Hematology/Oncology*.

[13] Fialkow PJ, Klein E, Klein G (1973). Immunoglobulin and glucose 6 phosphate dehydrogenase as markers of cellular origin in Burkitt lymphoma. *Journal of Experimental Medicine*.

[14] Burkitt D (1967). Long-term remissions following one and two-dose chemotherapy for African lymphoma. *Cancer*.

[15] Clifford P, Singh S, Stjernswärd J, Klein G (1967). Long-term survival of patients with Burkitt’s lymphoma: an assessment of treatment and other factors which may relate to survival. *Cancer Research*.

[16] Morrow RH, Pike MC, Kisuule A (1967). Survival of Burkitt’s lymphoma patients in Mulago Hospital, Uganda. *British Medical Journal*.

[17] Ziegler JL (1972). Chemotherapy of Burkitt’s lymphoma. *Cancer*.

[18] Ziegler JL, Bluming AZ, Fass L, Morrow RH (1972). Relapse patterns in Burkitt’s lymphoma. *Cancer Research*.

[19] Burchenal JH (1966). Geographic chemotherapy—Burkitt’s tumor as a stalking horse for leukemia: presidential address. *Cancer Research*.

[20] Clift  RA, Wright DH, Clifford P (1963). Leukemia in Burkitt’s lymphoma. *Blood*.

[21] Magrath LT, Ziegler JL (1980). Bone marrow involvement in Burkitt’s lymphoma and its relationship to acute B-cell leukemia. *Leukemia Research*.

[22] Wright DH (1964). Burkitt’s tumour. A post-mortem study of 50 cases. *The British Journal of Surgery*.

[23] Anderson JR, Jenkin RDT, Wilson JF (1993). Long-term follow-up of patients treated with COMP or LSA2L2 therapy for childhood non-Hodgkin’s lymphoma: a report of CCG-551 from the Childrens Cancer Group. *Journal of Clinical Oncology*.

[24] Ziegler JL, Bluming AZ, Magrath IT, Carbone PP (1972). Intensive chemotherapy in patients with generalized Burkitt’s lymphoma. *International Journal of Cancer*.

[25] Olweny CLM, Katongole-Mbidde E, Otim D (1980). Long-term experience with Burkitt’s lymphoma in Uganda. *International Journal of Cancer*.

[26] Olweny CLM, Katongole Mbidde E, Kaddu Mukasa A (1976). Treatment of Burkitt’s lymphoma: randomized clinical trial of single agent versus combination chemotherapy. *International Journal of Cancer*.

[27] Olweny CLM, Atine I, Kaau Mukasa A (1977). Cerebrospinal irradiation of Burkitt’s lymphoma. Failure in preventing central nervous system relapse. *Acta Radiologica. Therapy, Physics and Biology*.

[28] Nkrumah FK, Perkins IV (1976). Burkitt’s lymphoma. A clinical study of 110 patients. *Cancer*.

[29] Nkrumah FK, Perkins IV, Biggar RJ (1977). Combination chemotherapy in abdominal Burkitt’s lymphoma. *Cancer*.

[30] Ziegler JL, Bluming AZ (1971). Intrathecal chemotherapy in Burkitt’s lymphoma. *British Medical Journal*.

[31] Ziegler JL, Magrath IT, Olweny CLM (1979). Cure of Burkitt’s lymphoma. Ten-year follow-up of 157 Ugandan patients. *Lancet*.

[32] Haddy TB, Adde MA, Magrath IT (1991). CNS involvement in small noncleaved-cell lymphoma: is CNS disease per se a poor prognostic sign?. *Journal of Clinical Oncology*.

[33] Magrath IT, Lwanga S, Carswell W, Harrison N (1974). Surgical reduction of tumour bulk in management of abdominal Burkitt’s lymphoma. *British Medical Journal*.

[34] Norin T, Clifford P, Einhorn J (1971). Conventional and superfractionated radiation therapy in Burkitt’s lymphoma. *Acta Radiologica: Therapy, Physics, and Biology*.

[35] Ziegler JL (1977). Treatment results of 54 American patients with Burkitt’s lymphoma are similar to the African experience. *New England Journal of Medicine*.

[36] Reiter A, Schrappe M, Tiemann M (1999). Improved treatment results in childhood B-cell neoplasms with tailored intensification of therapy: a report of the Berlin-Frankfurt-Munster group trial NHL-BFM 90. *Blood*.

[37] Patte C, Auperin A, Michon J (2001). The Société Française d'Oncologie Pédiatrique LMB89 protocol: highly effective multiagent chemotherapy tailored to the tumor burden and initial response in 561 unselected children with B-cell lymphomas and L3 leukemia. *Blood*.

[38] Hoelzer D, Ludwig WD, Thiel E (1996). Improved outcome in adult B-cell acute lymphoblastic leukemia. *Blood*.

[39] Blum KA, Lozanski G, Byrd JC (2004). Adult Burkitt leukemia and lymphoma. *Blood*.

[40] Magrath I, Adde M, Shad A (1996). Adults and children with small non-cleaved-cell lymphoma have a similar excellent outcome when treated with the same chemotherapy regimen. *Journal of Clinical Oncology*.

[41] Adde M, Shad A, Venzon D (1998). Additional chemotherapy agents improve treatment outcome for children and adults with advanced B-cell lymphomas. *Seminars in Oncology*.

[42] Meinhardt A, Burkhardt B, Zimmermann M (2010). Phase II window study on rituximab in newly diagnosed pediatric mature B-cell non-Hodgkin’s lymphoma and Burkitt leukemia. *Journal of Clinical Oncology*.

[43] Linch DC Burkitt lymphoma in adults.

[44] Mohamedbhai SG, Sibson K, Marafioti T (2011). Rituximab in combination with CODOX-M/IVAC: a retrospective analysis of 23 cases of non-HIV related B-cell non-Hodgkin lymphoma with proliferation index >95%. *British Journal of Haematology*.

[45] Hesseling PB, Broadhead R, Molyneux E (2003). Malawi pilot study of Burkitt lymphoma treatment. *Medical and Pediatric Oncology*.

[46] Kazembe P, Hesseling PB, Griffin BE, Lampert I, Wessels G (2003). Long term survival of children with burkitt lymphoma in Malawi after cyclophosphamide monotherapy. *Medical and Pediatric Oncology*.

[47] Hesseling P, Broadhead R, Mansvelt E (2005). The 2000 burkitt lymphoma trial in Malawi. *Pediatric Blood and Cancer*.

[48] Harif M, Barsaoui S, Benchekroun S (2008). Treatment of B-cell lymphoma with LMB modified protocols in Africa—report of the French-African Pediatric Oncology Group (GFAOP). *Pediatric Blood and Cancer*.

[49] Hesseling P, Molyneux E, Kamiza S, Israels T, Broadhead R (2009). Endemic Burkitt lymphoma: a 28-day treatment schedule with cyclophosphamide and intrathecal methotrexate. *Annals of Tropical Paediatrics*.

[50] Hesseling PB, Molyneux E, Kamiza S, Broadhead R (2008). Rescue chemotherapy for patients with resistant or relapsed endemic Burkitt’s lymphoma. *Transactions of the Royal Society of Tropical Medicine and Hygiene*.

[51] Gad-El-Mawla N, Hamza MR, Abdel-Hadi S (1991). Prolonged disease-free survival in pediatric non-Hodgkin’s lymphoma using ifosfamide-containing combination chemotherapy. *Hematological Oncology*.

[52] Advani S, Pai S, Adde M (1997). Preliminary report of an intensified, short duration chemotherapy protocol for the treatment of pediatric non-Hodgkin’s lymphoma in India. *Annals of Oncology*.

[53] Magrath I (2009). Lessons from clinical trials in African Burkitt lymphoma. *Current Opinion in Oncology*.

[54] Magrath IT (2006). Treatment of Burkitt lymphoma in children and adults: lessons from Africa. *Current Hematologic Malignancy Reports*.

